# Proton Beam Therapy for a Rare Anaplastic Pleomorphic Xanthoastrocytoma: Case Report and Literature Review

**DOI:** 10.1016/j.ijpt.2024.100736

**Published:** 2024-12-25

**Authors:** Wei Han, Yonglong Jin, Jie Wang, Shuyan Zhang, Shigeharu Hashimoto, Zishen Wang, Wei Wang, Yinuo Li, Weiwei Wang, Lu Yang, Shosei Shimizu

**Affiliations:** 1 Department of Pediatric Oncology, Beijing Children's Hospital, Capital Medical University, Beijing China; 2 Department of Radiotherapy, The Affiliated Hospital of Qingdao University, Qingdao, China; 3 School of Public Health, Qingdao University, Qingdao, China; 4 Department of Radiotherapy Physics and Technology, Hebei Yizhou Cancer Hospital, Zhuozhou, China; 5 Department of Pediatric Radiation Therapy Center/Pediatric Proton Beam Therapy Center, Hebei Yizhou Cancer Hospital, Zhuozhou, China; 6 Department of Radiology, Hebei Yizhou Cancer Hospital, Zhuozhou, China; 7 Department of Radiation Oncology, University of Tsukuba, Tsukuba, Ibaraki, Japan

**Keywords:** Anaplastic pleomorphic xanthoastrocytoma, Proton beam therapy, Radiation therapy, High-grade glioma, Pediatric

## Abstract

Anaplastic pleomorphic xanthoastrocytoma (PXA) is a rare, aggressive WHO grade III tumor that primarily affects children and young adults. Despite surgery being the primary treatment, achieving complete tumor removal is often difficult due to its infiltrative nature, necessitating additional therapies like proton beam therapy (PBT). PBT, known for its precision in targeting tumors while minimizing damage to surrounding healthy tissue, has shown promise in treating malignant gliomas. We present the case of a 9-year-old girl with anaplastic PXA treated with PBT following incomplete surgical resection. A total dose of 60 Gy (RBE) in 15 fractions was administered, leading to significant tumor reduction, no progression, and improved local control at the 1-year follow-up, with no observed adverse effects. Based on short-term follow-up results, our study highlights the potential of PBT in managing anaplastic PXA, demonstrating favorable local outcomes and a low incidence of radiation-induced complications. While long-term follow-up and evaluation are necessary to further support these findings, this case represents only the second reported instance of anaplastic PXA treated with PBT, contributing to the growing body of evidence supporting its efficacy in this rare tumor type.

## Introduction

Pleomorphic xanthoastrocytoma (PXA) is a rare primary brain tumor that predominantly affects children and young adults.^1^ Classified by the World Health Organization (WHO) as a grade II tumor, PXA generally exhibits relatively indolent behavior and favorable prognosis following surgical resection.^2^ This type of tumor typically occurs in the temporal lobe and supratentorial compartment.^3^ However, a subset of these tumors demonstrate more aggressive characteristics and are classified as anaplastic PXAs (WHO grade III). These high-grade PXAs pose significant treatment challenges due to their increased proliferation, higher recurrence rates, and potential for malignant transformation.^4^

Anaplastic PXA is histologically distinguished by increased cellularity, pleomorphism, and mitotic activity, alongside features such as necrosis and endothelial proliferation.^5^ These histopathological hallmarks not only contribute to its aggressive clinical course but also necessitate a more rigorous therapeutic approach. Despite surgical resection being the cornerstone of treatment, complete removal is often impeded by the tumor’s location and infiltrative nature, particularly when it involves critical brain regions.^6^

Adjuvant therapies, including radiation therapy (RT), play crucial roles in managing residual or recurrent anaplastic PXA.^7^ Proton beam therapy (PBT), due to its superior dosimetric properties, has emerged as a promising modality in the treatment of various high-grade gliomas.^8^, ^9^ PBT offers the advantage of precise tumor targeting while minimizing radiation exposure to surrounding healthy tissues, thus reducing the risk of treatment-related morbidity.^10^, ^11^

This case report aims to discuss the postoperative PBT in a pediatric patient with WHO grade III anaplastic PXA, emphasizing the potential of this new treatment approach. By presenting detailed clinical and radiographic data, this report seeks to contribute to the growing body of literature on anaplastic PXAs and highlight the benefits of incorporating PBT into the therapeutic regimen for such challenging tumors.

## Case presentation

**Figure 1:**
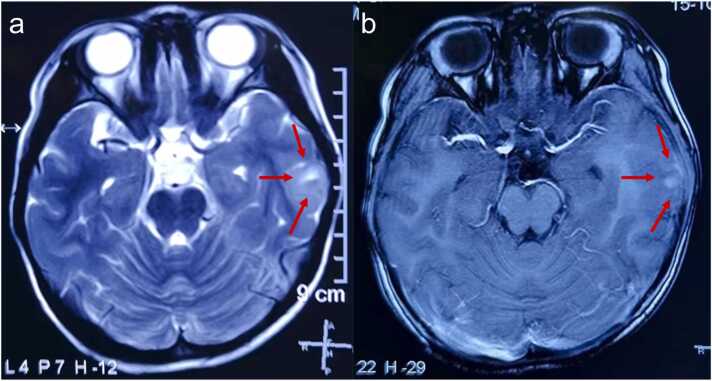
Cranial magnetic resonance imaging 20 months prior to surgery. (a) Axial T2WI: The red arrows highlighted a hyperintense mass in the left temporal lobe with an irregular shape. Mild edema was observed surrounding the lesion. (b) Axial postcontrast T1WI: The same lesion seen in image a showed slight enhancement after contrast administration, with areas of irregular contrast uptake, measuring approximately 18 × 15 × 24 mm. There was mild displacement of the midline structure.

A 9-year-old girl initially presented to a local hospital 3 years ago with febrile convulsions. At that time, cranial magnetic resonance imaging (MRI) revealed a faintly hyperintense lesion measuring approximately 18 × 15 × 24 mm in the left temporal lobe (Figure 1). The lesion was suspected to be benign due to faint enhancement, and the patient opted for further follow-up.

**Figure 2:**
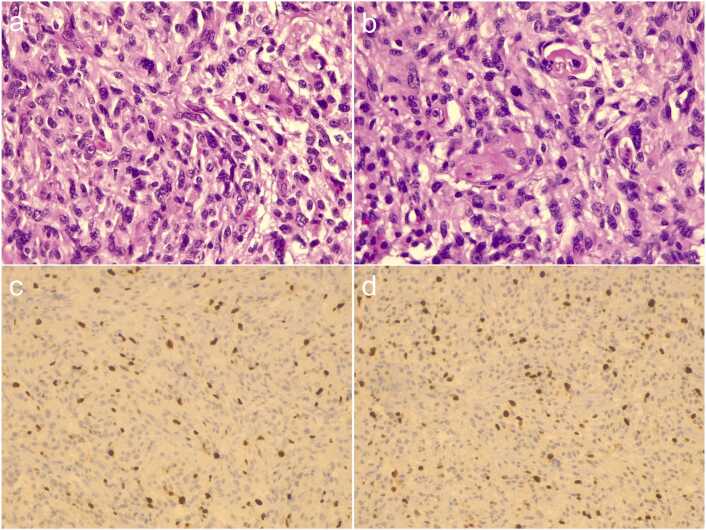
Postoperative pathological and immunohistochemical staining images of anaplastic PXA. (a and b) Pleomorphic and xanthic astrocytic cells, local epithelioid cells, multinucleated giant cells, and microvascular proliferation were observed in the surrounding brain parenchyma and perivascular spaces. The mitotic index was significantly elevated (more than 5 mitoses per 10 HPFs). (c and d) Immunohistochemical staining revealed a markedly increased Ki-67 labeling index. HPFs, high-power fields.

After 18 months, her headache symptoms worsened, and MRI at that time showed an increase in the size of the lesion in the left temporal region. Subsequently, she underwent a left frontotemporal craniotomy with nearly total resection of the lesion. The postoperative pathology and immunohistochemistry confirmed a diagnosis of anaplastic PXA, WHO grade III, with calcification (Figure 2). Under the microscope, some regions exhibited typical histological features of PXA, while other areas displayed anaplastic characteristics. Pleomorphic and xanthic astrocytic cells, local epithelioid cells, multinucleated giant cells, and microvascular proliferation were observed in the surrounding brain parenchyma and perivascular spaces. The mitotic index was significantly elevated (more than 5 mitoses per 10 high-power fields), as shown in Figure 2a and 2b. Immunohistochemical staining revealed a markedly increased Ki-67 labeling index (Figure 2c and 2d), with specific findings as follows: Ki-67 (5%-12%), GFAP (+), Olig-2 (+), p53 (+), ATRX (+), IDH1 R132H (-), MTAP (loss of expression), p16 (focally +), CD34 (+), BRAF V600E (+), Syn (+), NeuN (scattered +). The staining of the second slide showed Ki-67 (3%-12%) and CD68 (scattered +), while reticulin staining was positive on special chromosomal analysis. Given this combination of these findings, especially the BRAFV 600E mutation and the higher proliferation index Ki-67, the diagnosis was highly suggestive of anaplastic PXA.

**Figure 3:**
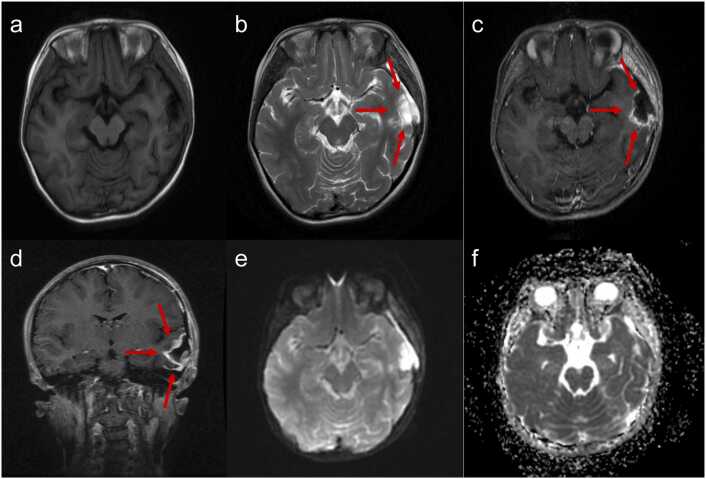
Cranial magnetic resonance imaging 1 month after surgery. An irregular surgical cavity change was observed in the left temporal lobe, measuring 41 × 28 × 52 mm. (a) Axial T1WI: The surgical cavity showed hypointense signal. (b) Axial T2WI: The surgical cavity showed hyperintense signal in the surrounding tissue (indicated by red arrows), with mild displacement of adjacent structures. (c and d) Axial and coronal postcontrast T1 images: The edges of the surgical cavity showed marked ring-like enhancement with an irregular shape. (e and f) Diffusion-weighted imaging and apparent diffusion coefficient maps: The lesion exhibited hyperintense signal in diffusion-weighted imaging and reduced signal in apparent diffusion coefficient maps, respectively, indicating the restricted diffusion.

One month postoperatively, the patient’s MRI demonstrated irregular surgical cavity changes in the left temporal lobe following tumor resection (Figure 3). On T1WI, the area appeared hypointense, while on T2WI, it showed hyperintensity. Postcontrast images displayed prominent ring-like enhancement at the resection margins, measuring 41 × 28 × 52 mm, indicating some tumor cells residues (Figure 3c and 3d). Diffusion-weighted imaging (DWI) revealed hyperintensity in both the resection site and surrounding margins (Figure 3e), with a corresponding reduced signal on apparent diffusion coefficient (ADC) maps (Figure 3f), indicating the restricted diffusion.

Given the high-grade malignancy and recurrence rate of the anaplastic PXA (CNS WHO grade III) and the need for definitive adjuvant therapy, the patient was referred to our hospital to undergo PBT.

Upon admission, the physical examination showed that the patient was oriented. A well-healed arcuate surgical scar was noted over the left temporal region. There were no abnormal neurological findings. Gross hearing examination revealed no abnormalities, and muscle strength was normal in all 4 limbs.

## Proton beam therapy

The patient underwent PBT in order to maximize tumor control and reduce recurrence while minimizing potential growth impairment in children.

**Figure 4:**
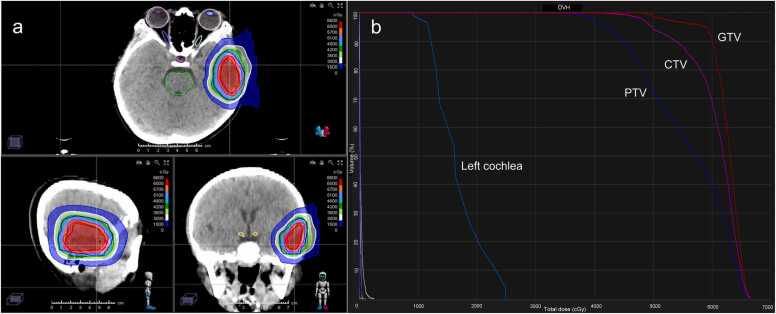
Dose distribution (a) and dose-volume histogram (b) of PBT. (a) The total dose of PBT was 60 Gy (RBE) in 15 fractions. The color wash indicated the dose distribution, with the highest dose regions centered on the GTV and the dose gradually decreasing outward. (b) The GTV received the highest dose coverage, followed by CTV and PTV. The left cochlea received a lower dose, as indicated by the steep drop-off in the dose-volume histogram curve, demonstrating dose sparing for critical structures. Red line: GTV; Pink line: CTV; Dark blue line: PTV; Light blue line: Left cochlea. Abbreviations: CTV, clinical target volume; GTV, gross tumor volume; and PTV, planning target volume.

Pencil beam scanning (PBS) intensity-modulated proton therapy (IMPT) is an advanced delivery technique in PBT. In PBS, a highly focused proton beam, only a few millimeters in diameter, is scanned across the tumor in a layer-by-layer approach, ensuring precise coverage of the tumor volume. The intensity of the proton beam is modulated to optimize the dose distribution within the tumor while minimizing exposure to surrounding healthy tissue. This technique is particularly advantageous for treating irregularly shaped or complex tumors, such as those located in the brain, and provides superior conformality compared to conventional photon-based intensity-modulated RT.^12^, ^13^ Although, in this case, the tumor was not located close to critical organs at risk (OARs), such as the brainstem, the protective effect of PBT on normal brain tissue in pediatric patients was considered. Compared to intensity-modulated RT, PBT generally delivers a lower dose to normal brain tissue, and the volume of tissue exposed to low-dose irradiation is significantly reduced. In this case, PBS IMPT was employed to deliver high radiation doses precisely to the tumor. Figure 4 shows the dose distribution of PBT.

In this study, the tumor’s aggressive nature promoted the consideration of a relatively rapid dose escalation to achieve local control. Additionally, prolonged treatment schedules were deemed impractical due to challenges with patient compliance. A shorter treatment duration of 3 weeks helped alleviate the burden on patients and their families. Aggressive schedules, 60 Gy (RBE)/15 fractions, were employed. The pretreatment positioning for PBT was performed. The patient was placed in a supine position with a head mask and a head-neck-shoulder board for immobilization. A computed tomography scan was conducted with a 2.5 mm slice thickness, covering the area from the vertex to the clavicles, and the images were uploaded to the treatment planning system. Radiation oncologists then developed the PBT plan. The gross tumor volume (GTV) for PBT was defined as the contrast-enhanced region on MRI. Clinical target volume was defined as the area surrounding the enhanced tumor, with a 5 to 10 mm margin added to the GTV. The planning target volume (PTV) was further expanded and included an additional nonuniform margin of 3 mm. A total prescribed dose of 60 Gy (RBE) in 15 fractions was delivered to the GTV, with 1 fraction administered daily. The dose for clinical target volume was 50 Gy (RBE)/15 fractions, and for PTV was 40 Gy (RBE)/15 fractions. The skin max dose was set to lower than 40 Gy (RBE). The number of ports was 3, and the robustness evaluation was based on the worst-case scenario. The dose requirements included GTV coverage ≥97%, with a setup uncertainty of 3 mm and a range uncertainty of 3.5%. For the IMPT plan, the maximum dose points for all OARs were almost below 1 Gy (RBE). Figure 4b demonstrates the dose-volume histogram of PBT. Throughout the radiation treatment, edema treatment was initiated starting from the ninth radiation fraction as part of intracranial pressure-lowering, administered alongside the increasing radiation dose, including 100 mL of 20% mannitol administered once daily and 1 mg of dexamethasone sodium phosphate intravenously once daily. The overall treatment proceeded smoothly and successfully, with no radiation-related acute adverse effects observed. Peripheral blood count results during and after irradiation showed no significant abnormalities.

## Evaluation of treatment outcomes

**Figure 5:**
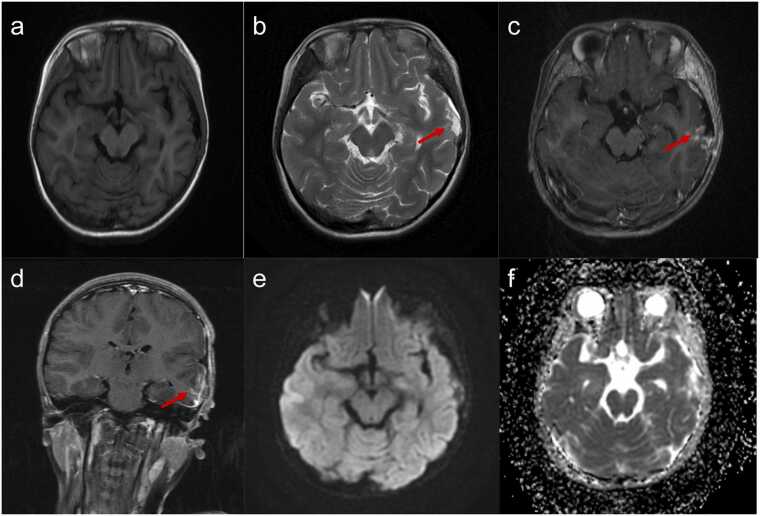
Cranial magnetic resonance imaging 1 year after proton beam therapy. (a) Axial T1WI and (b) T2WI: The postsurgical cavity has narrowed. (c) Axial and (d) coronal T1 postcontrast images: The ring-like enhancement around the lesion has significantly weakened, becoming spotty. (e) Diffusion-weighted imaging: The lesion signal has changed from hyperintense to hypointense. (f) Apparent diffusion coefficient maps: The lesion exhibited increased signal in apparent diffusion coefficient maps.

One year after PBT, compared to the pre-PBT MRI (Figure 3), the current MRI reveals a narrowing of the postsurgical cavity (Figure 5a and 5b) and no tumor progression. The contrast enhancement at the lesion has significantly weakened, leaving only a few spotty enhancements (Figure 5c and 5d). On DWI, the lesion area displays low signal intensity, indicating less restricted diffusion, which suggests that the number of tumor cells has decreased or that necrosis has occurred post-PBT (Figure 5e). Additionally, an increase in ADC values in the tumor region further indicates reduced tumor viability or increased necrosis (Figure 5f). Overall, the reduction in enhancement size and intensity, alongside the increased ADC values and changes in DWI, is indicative of the losing activity of tumor cells, supporting the efficacy of PBT. Additionally, the patient’s blood count, liver function, and kidney function remained within normal limits, with no adverse effects observed from PBT or the medication used to lower intracranial pressure. The patient’s hearing examination revealed no abnormalities.

As the effects of RT may continue to manifest over several years following treatment,^14^, ^15^ ongoing follow-up observations for this patient will be conducted in the future.

## Discussion

### Clinical features of PXAs

PXA, WHO grade II tumor, is a rare type of glioma, accounting for approximately 1% of all astrocytomas.^16^ It typically presents with symptoms such as headache, seizures, sensory abnormalities (localized neurological deficits), nausea, and vomiting (due to increased intracranial pressure).^17^ Among PXAs, a more aggressive variant known as anaplastic PXA is classified as a grade III tumor, characterized by higher mitotic activity, increased cellularity, and a poor prognosis.^18^ According to data from the National Institutes of Health, PXA can occur at any age but is most common in young adults (over 20 years old), with a slightly higher incidence in females than in males. The overall 5-year survival rate is 70.4%. However, the study showed that WHO grading significantly influences overall survival; in a cohort of 62 patients, the 5-year overall survival was significantly different between PXA and anaplastic PXA (80.8% vs 47.6%; *P* = .0009).^19^

### Absence of standardized treatment regimen for PXAs

Standard treatment regimens for PXAs, particularly for cases exhibiting anaplastic transformation from the outset, are not well established.^6^ The progression of PXAs from grade II to grade III indicates higher aggressiveness and an increased risk of recurrence.^19^ The findings of Zuo et al^20^ confirmed that, among 56 patients with high-grade PXA, the recurrence rate was 70% after a median follow-up of 35 months. Another study indicated that the recurrence rates for patients with grade II and grade III PXA were 27.5% and 77.8% during the observation period, respectively.^1^ While gross total resection remains the primary treatment modality, achieving clear surgical margins is often challenging due to the infiltrative nature of anaplastic PXA. Given the aggressive behavior of anaplastic PXA, radical adjuvant therapy following surgical resection is crucial for improving patient outcomes. Although chemotherapy has been explored, there is limited evidence supporting its efficacy in anaplastic PXA, particularly for long-term disease control.^7^ In the study by Zhu et al,^6^ tumor recurrence and distant dissemination were observed despite the continuous use of Temozolomide. This raises doubts about the benefits of Temozolomide in the treatment of anaplastic PXA.

The evaluation of the role of RT in the treatment of PXA is limited due to the lack of extensive data. Many scholars suggest considering adjuvant or salvage RT in cases of recurrence, residual, or metastatic disease.^21^ The study by Patibandla et al^22^ noted that chemotherapy, including Temozolomide, is ineffective for treating anaplastic PXA, but stereotactic radiosurgery may help in halting disease progression, warranting further research. Additionally, studies have reported that postoperative RT is associated with improved progression-free survival, especially in cases of subtotal resection and/or anaplastic PXA, where treatment outcomes tend to be poor, suggesting early RT should be considered.^7^ As demonstrated in this case, the initial tumor presentation was misleading, as the MRI showed only faint enhancement, leading to a misdiagnosis as a more benign condition. However, the lesion was ultimately diagnosed as grade III anaplastic PXA, and postoperative MRI revealed residual tumor cells at the resection margins due to the invasion nature of the anaplastic PXA, underscoring the necessity of adjuvant RT.

### Proton beam therapy—new therapeutic weapon for PXAs

For pediatric patients, clinicians must carefully weigh the potential negative side effects of brian RT, such as neurocognitive impairment, growth problems, and hormone deficiency.^23^ PBT has thus emerged as the preferred adjuvant modality in such pediatric cases, and it has been proven to reduce the risk of damage to developing vital organs and the risk of secondary malignancies.^24^, ^25^ PBT has garnered significant attention for its ability to precisely target tumors while minimizing radiation exposure to surrounding healthy tissues, which is a critical factor when treating brain tumors like anaplastic PXA. The unique dosimetric properties of protons allow for superior conformal treatment delivery, ensuring that high doses are concentrated within the tumor while sparing adjacent normal structures.^26^ This capability is particularly important when managing pediatric tumors or tumors located in delicate areas, as minimizing radiation exposure to developing tissues can prevent long-term complications.^27^, ^28^ The therapeutic efficacy of PBT in treating high-grade gliomas, including grade III tumors, has been well documented.^29^ Furthermore, when searching the keyword of “Proton therapy, anaplastic PXA” in a search engine, there is only 1 case report from the Mayo Clinic in 2016 that was found, detailing the first case of an adult woman treated with PBT for anaplastic PXA. Over a span of more than 20 years, she underwent 5 craniotomies, 3 rounds of chemotherapy, and 6 weeks of RT in 2007, but the tumor eventually recurred. Due to the increased sensitivity and risk of her brain tissue to a second round of RT, she was recommended for PBT. This case highlights how PBT offers a viable treatment option for aggressive, recurrent anaplastic PXA, particularly when other treatment modalities are limited.^30^

### PBS IMPT technology—more precision and higher dose in local

In this case, the PBS IMPT was utilized. IMPT allows for the use of PBS systems to achieve particularly steep dose gradients, further enhancing the advantages of PBT. Historically, protons were delivered through passive scattering systems, with the distal end of the proton beam controlled or shaped by using compensators. In contrast, spot scanning proton therapy, or what is now referred to as PBS, involves scanning a fine proton pencil beam at various energies within the target volume to achieve a more precise dose distribution at the required depth.^31^ A study from a US group demonstrated that, during craniospinal irradiation in brain tumor patients, PBS was more effective in sparing the cochlea and lenses compared to passive scattering delivery.^32^ Additionally, for patients requiring postoperative RT after endoscopic resection of skull base tumors, PBS IMPT was shown to better protect critical OARs compared to intensity-modulated photon RT.^33^ For this case, the tumor was located in the temporal lobe, not very close to the brainstem or optic chiasm. The advantages of IMPT in protecting OARs were not fully apparent compared to passive scatter PBT. However, considering dose fall-off, the sharp lateral fall-off in IMPT can reduce the risk of underdosing at the tumor margins. Additionally, in passive scattering, the skin dose tends to be higher than in IMPT. Therefore, overall, IMPT still provided certain advantages for this case.

The decision to use 60 Gy (RBE)/15 fractions was reached after multiple discussions with the patient’s guardians. First, the tumor’s highly aggressive nature prompted us to consider a relatively rapid dose escalation to achieve local control. Additionally, due to challenges with patient compliance, a prolonged treatment schedule was deemed impractical. A shorter treatment duration of 3 weeks would help alleviate the burden on the patient and their family. In this case, the lesion was located at the margin of the temporal lobe, and the dose distribution advantages of PBT (deliver a sharp dose fall-off and effectively spare surrounding normal tissues) allowed the radiation to normal brain tissue to be minimized. As previously mentioned, the tumor had a high local recurrence rate, and there was no evidence suggesting that recurrence could be controlled through standard fractionation. For this case, the prescribed dose to the PTV was 40 Gy (RBE), delivered in 15 fractions, with a minimum fraction dose of 2.6 Gy (RBE). However, as shown in the dose-volume histogram, the maximum dose to the PTV could exceed 4 Gy (RBE), indicating that associated safety concerns must be carefully considered, which was indeed implemented during the actual treatment. The risks and benefits of local control and potential adverse events were thoroughly discussed with the patient’s guardians. They were fully informed and accepted the possibility of side effects, such as brain necrosis associated with hypofractionation irradiation. In the worst-case scenario, even if brain necrosis occurred, the anatomical location of the lesion would allow surgical intervention. Ultimately, an aggressive treatment plan of 60 Gy (RBE)/15 fractions was adopted. However, it is still important to emphasize that the indication for a single large dose of radiation for pediatric patients should be carefully considered.

In this case, with 1 year of follow-up so far, long-term observation will be required. However, up to now, no adverse events, such as brain necrosis and hearing loss, have appeared on imaging or clinically, and local control has been achieved. This is a rare disease with limited information on effective treatments, but many reports emphasize the need for high-dose treatment while also protecting normal brain tissue. PBS IMPT may be suggested as a valuable treatment option in such cases.

This case further underscores the importance of integrating radical PBT into the postoperative treatment strategy for anaplastic PXA to achieve optimal local control and prevent recurrence. In this case, PBT was chosen to maximize tumor control while minimizing damage to adjacent critical structures and surrounding healthy tissues. Advanced techniques, PBS IMPT, further optimize dose distribution by allowing layer-by-layer modulation of the proton beam. This technique provides more conformal dose coverage compared to conventional photon therapy, particularly for irregularly shaped tumors like those found in anaplastic PXA cases. The results of this treatment show promising tumor control, with reduced post-treatment enhancement on MRI and decreased DWI signals, indicating tumor cell necrosis. We present the treatment process and short-term outcomes for this specific tumor type, exploring the potential application of PBT as a new therapeutic option for similar cases in the future. While we acknowledge that strong conclusions cannot be drawn from a single case report, this report provides preliminary insights into the application of PBT for this tumor type. It also underscores the need for further studies and extended follow-up to fully assess the efficacy of IMPT in similar cases.

## Conclusion

This is the second reported case that suggests the efficacy and safety of PBT for anaplastic PXA, and the first case reported in pediatric patients. The therapy achieved excellent local tumor control, with no side effects observed 1-year post-PBT. The precision of PBT in minimizing radiation exposure to surrounding healthy tissues was crucial in preserving function. These findings underscore PBT as a valuable option for managing anaplastic PXA, particularly in complex cases. Long-term follow-up will be essential to confirm sustained efficacy.

## Ethics approval

The present study was performed in accordance with the principles of the Declaration of Helsinki (2013 version). Approval was granted by the Ethics Committee of Hebei Yizhou Cancer Hospital (Zhuozhou, China).

## Author Contributions

Wei Han: Conceptualization, Resources, Writing- Original draft, Visualization. Yonglong Jin: Writing- Review and editing. Jie Wang: Software. Shuyan Zhang: Resources, Methodology, Visualization. Shigeharu Hashimoto: Writing- Review and editing. Zishen Wang: Software. Wei Wang: Visualization, Investigation, Validation. Yinuo Li: Writing- Review and editing. Weiwei Wang: Resources, Methodology, Visualization. Lu Yang: Resources, Investigation, Visualization. Shosei Shimizu (Qingshui Xiangxing): Conceptualization, Methodology, Validation, Writing- Review and editing, Visualization, Supervision, Project administration.

## Declaration of Conflicts of Interest

The authors declare that they have no known competing financial interests or personal relationships that could have appeared to influence the work reported in this paper.

## Data Availability

The data sets generated during and/or analyzed during the current study are available from the corresponding author upon reasonable request.
